# The Potential Impact of Male Circumcision on HIV in Sub-Saharan Africa

**DOI:** 10.1371/journal.pmed.0030262

**Published:** 2006-07-11

**Authors:** Brian G Williams, James O Lloyd-Smith, Eleanor Gouws, Catherine Hankins, Wayne M Getz, John Hargrove, Isabelle de Zoysa, Christopher Dye, Bertran Auvert

**Affiliations:** **1**World Health Organization, Stop TB Department, Geneva, Switzerland; **2**Department of Environmental Science, Policy, and Management, University of California Berkeley, Berkeley, California, United States of America; **3**Center for Infectious Disease Dynamics, Pennsylvania State University, University Park, Pennsylvania, United States of America; **4**Joint United Nations Programme on HIV/AIDS (UNAIDS), Policy, Evidence, and Partnerships Department, Geneva, Switzerland; **5**South African Centre for Epidemiological Modelling and Analysis, Stellenbosch, South Africa; **6**World Health Organization, Family and Community Health, Geneva, Switzerland; **7**INSERM, Saint Maurice, France; **8**University of Versailles-Saint Quentin, Faculté de Médecine Paris-Ile-de-France-Ouest, Saint Maurice, France; **9**Assistance Publique-Hôpitaux de Paris, Hôpital Ambroise Pare, Boulogne, France; San Francisco General HospitalUnited States of America

## Abstract

**Background:**

A randomized controlled trial (RCT) has shown that male circumcision (MC) reduces sexual transmission of HIV from women to men by 60% (32%−76%; 95% CI) offering an intervention of proven efficacy for reducing the sexual spread of HIV. We explore the implications of this finding for the promotion of MC as a public health intervention to control HIV in sub-Saharan Africa.

**Methods and Findings:**

Using dynamical simulation models we consider the impact of MC on the relative prevalence of HIV in men and women and in circumcised and uncircumcised men. Using country level data on HIV prevalence and MC, we estimate the impact of increasing MC coverage on HIV incidence, HIV prevalence, and HIV-related deaths over the next ten, twenty, and thirty years in sub-Saharan Africa. Assuming that full coverage of MC is achieved over the next ten years, we consider three scenarios in which the reduction in transmission is given by the best estimate and the upper and lower 95% confidence limits of the reduction in transmission observed in the RCT.

MC could avert 2.0 (1.1−3.8) million new HIV infections and 0.3 (0.1−0.5) million deaths over the next ten years in sub-Saharan Africa. In the ten years after that, it could avert a further 3.7 (1.9−7.5) million new HIV infections and 2.7 (1.5−5.3) million deaths, with about one quarter of all the incident cases prevented and the deaths averted occurring in South Africa. We show that a) MC will increase the proportion of infected people who are women from about 52% to 58%; b) where there is homogenous mixing but not all men are circumcised, the prevalence of infection in circumcised men is likely to be about 80% of that in uncircumcised men; c) MC is equivalent to an intervention, such as a vaccine or increased condom use, that reduces transmission in both directions by 37%.

**Conclusions:**

This analysis is based on the result of just one RCT, but if the results of that trial are confirmed we suggest that MC could substantially reduce the burden of HIV in Africa, especially in southern Africa where the prevalence of MC is low and the prevalence of HIV is high. While the protective benefit to HIV-negative men will be immediate, the full impact of MC on HIV-related illness and death will only be apparent in ten to twenty years.

## Editors' Summary

**Background.:**

Africa is the continent most affected by HIV/AIDS, and it is important to consider all possible means of reducing the spread of HIV infection. Male circumcision has been a tradition in many parts of Africa for hundreds of years. Boys who are circumcised usually have it done in late childhood or their early teenage years. It was noticed some years ago that those African groups in which circumcision is routinely done on all boys have fewer cases of HIV/AIDS than are found in groups where circumcision is not a tradition. This finding gave rise to the idea that circumcision might give a degree of protection against HIV, though it was recognised that some other, unknown difference between these groups of people might actually be the important factor. In 2005 a trial was reported from the Orange Farm area of South Africa, in which uncircumcised men were offered the chance to be circumcised. The men who agreed were divided at random into those who had the operation straightaway and those who were to have it two years later. During the next 18 months, the number of new cases of HIV infection was much higher amongst the men who had not been circumcised. Circumcision did therefore seem to offer a measure of protection against infection. This protective effect was estimated at being about 60%. Similar trials are under way in other parts of Africa but there are no results available from them at this stage.

**Why Was This Study Done?:**

If the level of effectiveness of circumcision suggested by the South African trial is correct, then, as one part of a range of measures to reduce the spread of HIV, it would seem logical to encourage the practice of male circumcision. It would be useful to have an estimate of just how many new cases could be prevented and how many lives would be saved by the promotion of male circumcision. Calculations would have to allow for various factors, such as the present level of HIV infection, which varies from one country to another, and the fact that many men are already circumcised.

**What Did the Researchers Do and Find?:**

This research did not involve collecting any new data. The researchers used mathematical modelling to make calculations. They based their model on data from the Orange Farm trial and on information from various sub-Saharan African countries on the proportion of men who are circumcised and the proportion who are HIV-positive. They made the assumption that if circumcision is intensively promoted, all men in those countries will be circumcised in 10 years time. They calculated the number of new cases that would be prevented and the lives that would be saved in ten years, 20 years, and 30 years time. Their best estimate is that with the promotion of male circumcision, two million cases and 0.3 million deaths will be avoided in ten years time. Over the following ten years, according to the researchers' model, a further 3.7 million cases and 2.7 million deaths would be prevented. Most of the initial impact would be in men, but the reduction in the number of HIV-positive men would in time also lower the risk of women becoming infected. Overall, on the basis of these calculations, male circumcision would reduce the rate of infections by about 37%—both female-to-male and male-to-female transmission. The size of the impact would vary from one country to another; it would be greatest in southern Africa where HIV infection rates are high and circumcision rates relatively low compared with the rest of sub-Saharan Africa.

**What Do These Findings Mean?:**

Male circumcision alone cannot bring the HIV/AIDS epidemic in Africa under control. Even circumcised men can become infected, though their risk of doing so is much lower. However, the researchers call for the promotion of male circumcision to become a major part of AIDS control programmes. Their results are based on the findings of just one study (the Orange Farm trial), and it will be important to repeat the calculations when further studies have been completed.

**Additional Information.:**

Please access these Web sites via the online version of this summary at http://dx.doi.org/10.1371/journal.pmed.0030262:

• The Orange Farm trial was published in *PLoS Medicine*. Several articles discussing the trial were also published in the same issue of the journal

• The Joint United Nations Programme on HIV/AIDS (UNAIDS) has information about the state of the HIV/AIDS epidemic and prevention strategies worldwide. It produces an annual report and has documents on a wide range of topics

• AEGIS is the world's largest searchable database on HIV and AIDS.

• Many organizations provide information on AIDS prevention—for example, the Terrence Higgins Trust

• The World Bank's Global HIV/AIDS Program has a report about male circumcision and HIV infection

## Abstract 3

A modelling study, based on one trial plus national figures for current prevalence of HIV and of male circumcision (MC), found increasing MC in Africa could produce major fall in HIV prevalence in 10-12 years.

## Introduction

Ambitious targets have been set for the control of the HIV pandemic [ 1– 3]. While access to life-saving anti-retroviral therapy (ART) for those infected with HIV is increasing rapidly throughout the world, effective programmes to reduce HIV transmission are still needed [ 4], especially in Africa. Many factors influence the risk of acquiring and transmitting HIV [ 5], but where measures intended to reduce transmission have been rigorously tested at a population level, the results have been mixed [ 6, 7]. Furthermore, an effective vaccine will probably not be available for a decade or more [ 8].

**Figure 1:**
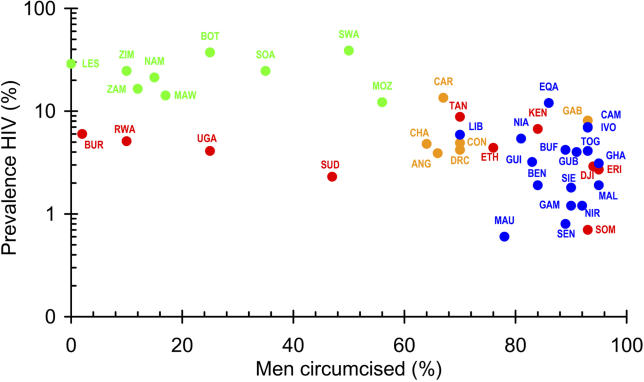
The Relationship between the Prevalence of HIV and MC in Sub-Saharan Africa The percent prevalence of HIV [ 12] is plotted on a logarithmic scale against the estimated proportion of adult men who are circumcised [ 32, 33]. Green, southern Africa; red, East Africa; orange, Central Africa; blue, West Africa. C, Central Africa; E, East Africa; S, South Africa; W, West Africa. ANG, Angola; BEN, Benin; BOT, Botswana; BUF, Burkina Faso; BUR, Burundi; CAM, Cameroon; CAR, Central African Republic; CHA, Chad; CON, The Congo; DJI, Djibouti; DRC, Democratic Republic of the Congo; EQA, Equatorial Guinea; ERI, Eritrea; ETH, Ethiopia; GAB, Gabon; GAM, Gambia; GHA, Ghana; GUB, Guinea Bissau; GUI, Guinea; IVO, Côte d'Ivoire; KEN, Kenya; LES, Lesotho; LIB, Liberia; MAL, Mali; MAU, Mauritania; MAW, Malawi; MOZ, Mozambique; NAM, Namibia; NIA, Nigeria; NIR, Niger; RWA, Rwanda; SEN, Senegal; SIE, Sierra Leone; SOA, South Africa; SOM, Somalia; SUD, Sudan; SWA, Swaziland; TAN, Tanzania; TOG, Togo; UGA, Uganda; ZAM, Zambia; ZIM, Zimbabwe.

There is now clear evidence that male circumcision (MC) significantly reduces female-to-male transmission of HIV. In the first randomized controlled trial (RCT) to report on MC, Auvert and colleagues [ 9] have shown that MC reduces transmission from women to men by 60% (32%−76%; unless otherwise stated ranges are 95% CI). An earlier meta-analysis of observational studies found an adjusted relative risk for HIV in circumcised men of 0.42 (0.34−0.54) [ 10], although a Cochrane review gave a more cautious interpretation [ 11]. In sub-Saharan Africa, estimates of HIV prevalence [ 12] ( Figure 1) are significantly associated with the estimated prevalence of MC (correlation coefficient, *ρ* = −0.61; *p* < 0.0001). In countries where fewer than 30% of men are circumcised, the median prevalence of HIV is 17% (IQR: 6% to 27%, *n* = 9 countries); where more than 90% of men are circumcised it is 2.9% (IQR: 1.5% to 5.5%, *n* = 13 countries). In a multi-centre study in Africa, Herpes simplex 2 and not being circumcised were independent risk factors for HIV [ 13]; the prevalence of HIV was negatively correlated with the proportion of men who were circumcised (correlation co-efficient, *ρ* = −0.85; *p* < 0.001) [ 14]. RCTs in Kenya [ 15] and Uganda [ 16] will provide further information on the impact of MC on HIV and in particular on the possible impact of MC on male-to-female transmission of HIV.

The impact of increasing MC coverage on HIV depends on the prevalence of HIV and of MC and we assess this impact using both static and dynamic models. For purposes of illustration, country estimates are aggregated to four regions, but we also present results for South Africa where MC could have the greatest impact and where the estimates of MC and HIV prevalence are reliable. Estimates are made of the reduction in incidence, prevalence, and deaths in each region. Assuming that the prevalence of MC is increased from present levels to full coverage (as defined in Protocol S1 and illustrated in Figure S1) in either 2010 or 2015, we determine the relative impact that MC will have on HIV incidence, prevalence, and related mortality among circumcised and uncircumcised men and on women over the next thirty years. We consider MC independently of the changes that might arise from the implementation of other effective prevention programmes in order to focus on the impact of increasing MC coverage.

## Methods

To estimate the potential impact of MC on HIV prevalence, we first use a static model to estimate Δ *J,* the reduction in the incidence of infection *J.* Using the result derived below ( Equations 8–10), the reduction in incidence, if all men were circumcised, would be



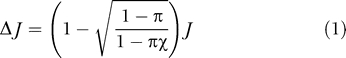



where *π* is the reduction in HIV transmission from women to men, χ is the proportion of men who are already circumcised, and the incidence is taken to be one-tenth of the current prevalence, corresponding to a mean life expectancy of ten years in the endemic state.

The reduction in incidence, calculated using Equation 1, gives an estimate of the impact of MC on the incidence of HIV but does not account for changes in the long-term dynamics of the epidemic. An immediate reduction in the rate of new infections will only be reflected in the prevalence of HIV and HIV-related deaths some years later, because prevalence depends on cumulative incidence and the mean time from infection to death is about ten years. For this reason, we also develop dynamic models to explore the impact of MC on incidence, prevalence, and deaths over several decades.

Although the models are general, we focus the discussion of the model development on parameter values pertaining to South Africa, which has the best national data on HIV and where MC is likely to have the biggest impact. We extrapolate to other countries by adjusting the relevant parameters to fit the data on national trends in HIV prevalence and the local prevalence of MC.

### Parameter Estimates for South Africa

Early in the epidemic, the prevalence of HIV in South Africa increased exponentially at a rate *r* = 0.55 ± 0.16/year, giving an intrinsic doubling time *d* = 1.26 ± 0.37 years [ 17]. The life expectancy after infection with HIV and without ART, standardized to a mean age at infection of 27 years, is *τ* = 9.8 ± 0.5 years [ 18, 19]. The average mortality rate *δ* = 1/τ is then 0.102 ± 0.005/year and the case reproduction number *R*_0_ = ( *r* + *δ*)/ *δ* is 6.4 ± 1.6 [ 20], where we have used Monte Carlo sampling from normal distributions to estimate the standard deviations [ 21].

### Asymmetry in Sexual Transmission

An infected man is about twice as likely, per contact, to infect a susceptible female partner, as an infected woman is to infect a susceptible male partner. In two European studies, the transmission ratio was 2.3 (1.1−4.8) [ 22] and 1.9 (1.1−3.3) [ 23], respectively, although the ratio was significantly lower for sex during menses and higher for anal sex and for sex involving older women [ 22]. The results of the RCT [ 9] suggest that MC will increase the transmission ratio by a further factor of 2.5 (1.5−4.2).

### Relative Prevalence of HIV in Men and in Women

To determine the relative prevalence of HIV in men and women as a function of the transmission parameters, we use a two-sex, susceptible–infected model. We let *i _f_* and *i _m_* be the prevalence of HIV in women and men. We assume that HIV-related mortality, *δ,* is independent of time since infection to facilitate the development of analytical expressions that can be used to parameterize the different effects on men and women in terms of an overall effect averaged over men and women. In the detailed analysis below ( Equations 12–15), we represent HIV-related mortality using a Weibull survival curve [ 24].

Although viral load is high during primary infection and near the end of life, we assume here that infectiousness is independent of time since infection. Then assuming an effective contact rate *c,* and probabilities of infection per contact of *φ _m_* for female-to-male transmission and *φ _f_* for male-to-female transmission, the model is



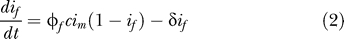





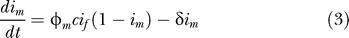



Early in the epidemic, *i _f_* and *i _m_* are both much less than 1, the initial growth rate is



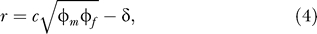



the ratio of the prevalence in women to that in men in the early stages of the epidemic is



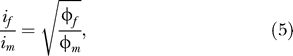



while the case reproduction number is



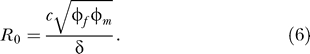



In the endemic state, the ratio of the prevalence in women to that in men is



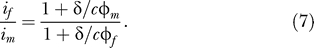



For the South African epidemic, if no men were circumcised, we estimate that *R*_0_ = 7.2 ± 1.8 so that with *δ* = 0.102 ± 0.005/y and *φ _f_ /φ _m_* = 2.0 ± 0.5 Equation 6 gives *cφ _m_*= 0.52 ± 0.16/year and *cφ _f_* = 1.04 ± 0.29/y. From Equation 7 the proportion of infected adults who are women is 52% ± 1%. (A more detailed examination of the proportion of infected adults who are women is given in Protocol S1 and Figure S2.)

### Collapsing the Two-Sex Model to a One-Group Model

To determine rates of infection, averaged over men and women, we replace Equations 2 and 3 by



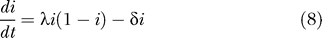



where *i* is the population prevalence averaged over men and women, and 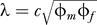 so that *R*_0_ for Equation 8 is the same as in Equation 6. It follows that reducing *φ _m_* by a factor of 1 − *π* is equivalent to reducing both *φ _m_* and *φ _f_* by a factor of 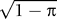.

To allow for the fact that not all men are circumcised, we let χ be the proportion of men who are circumcised and *π* be the reduction in female-to-male infectiousness when men are circumcised. Then assuming random mixing the average female-to-male infectiousness is reduced to



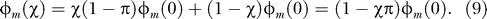



If the proportion of men who are already circumcised is *χ,* the effect of circumcising all men will be to reduce *φ _m_* to



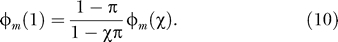



In Protocol S1 and Figures S4 and S5, we analyse a three-group model (distinguishing women, circumcised men, and uncircumcised men), and show that under certain assumptions it can be reduced to a one-group model with errors of less than 2.5% in the endemic prevalence for parameter values of interest.

### The Effect of Heterogeneity in Sexual Activity

Levels of sexual activity are highly skewed [ 25, 26]. If men and women have contact rate distributions with means and, and variances 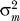 and 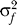, and mixing is random, then Equation 6 still holds [ 27] but with



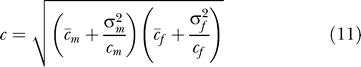



so that heterogeneity in sexual activity increases *R*_0_. However, MC only affects the transmission probabilities *φ _m_* and *φ _f_* and not the contact rates *c _m_* and *c _f_,* so that Equations 6–10 still hold.

With *R*_0_ ≈ 6, the predicted steady state prevalence, ( *R*_0_ − 1)/ *R*_0_ ≈ 83%, is much higher than is generally observed. In some models this is allowed for by assuming that people have either zero risk or a fixed risk, and then varying the risk-free fraction to fit the steady-state prevalence [ 28]. If we assume, more realistically, that there is a continuous distribution of risk, those at greater risk will tend to be infected earlier, and the risk of infection for uninfected people will fall as prevalence rises. Here we assume (and corroborate with South African data) that the average contact rate for those who are uninfected declines exponentially with prevalence (see Protocol S1 and Figure S6).

### The Aggregate Model

Based on the above arguments, we constructed an aggregate model in which we considered the HIV prevalence, incidence, and related deaths averaged over circumcised and uncircumcised men. The model was fitted to the available time series of HIV prevalence for each country and used to make projections. We let *S*( *t*) and *I*( *t*) be the number of susceptible and infected adults over the age of 15 years at time *t,* with *N*( *t*) = *S*( *t*) + *I*( *t*) *,* and *i*( *t*) = *I*( *t*)/ *N*( *t*). Adults enter the population at risk at a rate *b* times the population 15 years earlier so that the total population growth rate matches the reported values for each country [ 29]. To allow for heterogeneity in sexual behaviour and to fit the observed asymptotic prevalence of infection, the transmission parameter takes the value *λ*_0_ at the start of the epidemic and declines exponentially at rate *α* times the prevalence of infection. The background death rate is *μ*. We use a Weibull survivorship, *W*( *t*) *,* with a median of 9.8 years and a shape parameter of 2.25 to capture the dependence of HIV-related mortality on time since infection [ 24]. The model is then



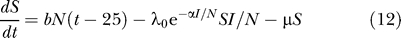





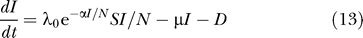





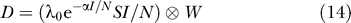



where o× indicates convolution (see Protocol S1) and *D* is number of HIV-related deaths per unit time. For those few countries, such as Uganda, where there is evidence that the prevalence of HIV is falling, we allow the transmission parameter to decline with mortality, *D/N,* to account for behavioural changes in response to the epidemic. To do this, we multiply *λ*_0_ in Equations 12–14 by e *^−ɛD/N^* and vary *ɛ* to fit the data.

The dynamic model was fitted to the UNAIDS estimates of national HIV prevalence in adults by least-squares. Epidemics were projected 30 years into the future assuming that the coverage of MC increases logistically (see Protocol S1 and Figure S1) from present levels to full coverage over ten years. Changing MC coverage is allowed for by scaling *λ*_0_ as in Equations 9 and 10. Three scenarios were considered for the impact of MC on female-to-male transmission of HIV corresponding to the best estimate and to the lower and upper 95% confidence limits from the RCT [ 9].

### Data

The data on circumcision ( Table 1) are taken from a study by Murdock in 1967 [ 30] updated by Bongaarts et al. in 1989 [ 31] and adjusted for ethnic groups by Wendell and Werker in 2004 [ 32]. We updated these estimates further using data from Demographic and Health Surveys (DHS) carried out by Macro International (Calverton, Maryland, United States) in Kenya, Tanzania, Mozambique, Uganda, Burkina Faso, Cameroon, and Ghana [ 33], and on national population–based surveys carried out in Botswana [ 34] and South Africa [ 35]. A study in Tanzania [ 36] where 41% of men were circumcised, found that self-reported MC had a sensitivity of 94% and a specificity of 72%, so self-reported rates may overestimate true rates. The most important change to the earlier data arises from the assumption that men in a particular ethnic group, bordering Ghana and Côte d'Ivoire, are not circumcised. The DHS survey for Ghana suggests that 95% of men are circumcised in contrast to the earlier estimate of 42% [ 32]. A study carried out in Abidjan [ 37] suggests that in Côte d'Ivoire the true rate of circumcision is 93%. We used these values here. The estimates of HIV prevalence are from UNAIDS [ 12], the estimates of population size and growth rates are from the United Nations Development Programme [ 29]. Data for the time trend in the prevalence of HIV in South Africa were obtained from the series of 14 annual antenatal clinic surveys [ 38].

**Table 1:**
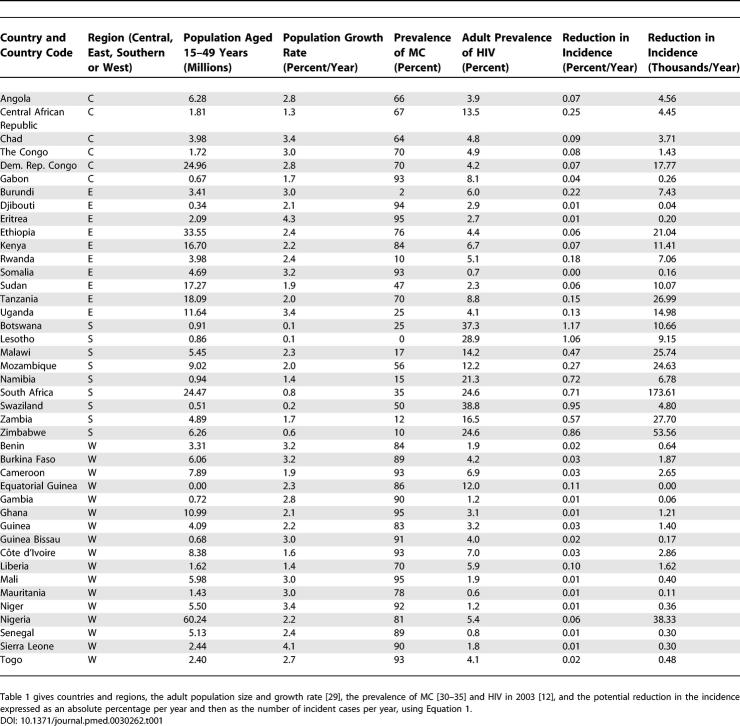
The Potential Reduction in HIV Incidence for Countries in Sub-Saharan Africa

## Results

**Figure 2:**
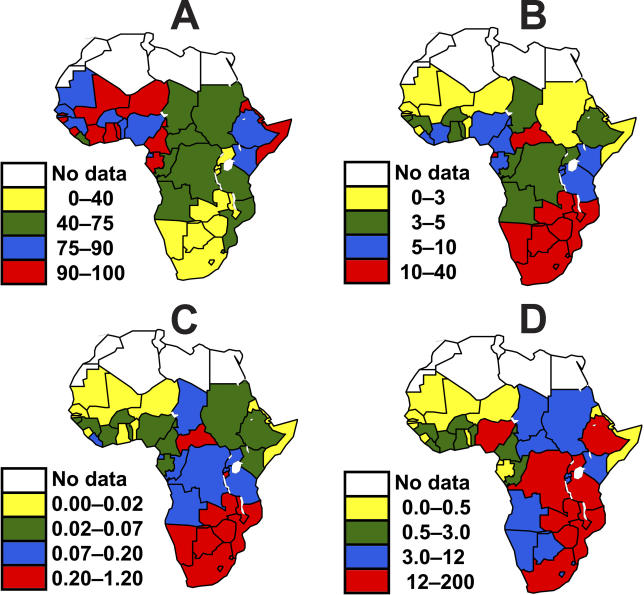
The Geographical Distribution of MC, HIV Prevalence, and the Potential Reduction in HIV Incidence if All Men Were Circumcised (A) Proportion of men who are circumcised, *χ* (%). (B) Prevalence of HIV in 2003, *P* (%). (C) Potential impact of MC on the incidence of HIV infection, Δ *J* (% per year), calculated using Equation 1. (D) Potential reduction in the number of new adult infections each year Δ *JN*, where *N* is the adult population (thousands per year). The incidence, *J*, is taken to be one-tenth of the prevalence, *P*. The data on which these maps are based are given in Table 1. *Disclaimer: The designations used and the presentation of material in Figure 2 do not imply the expression of any opinion whatsoever on the part of the World Health Organization concerning the legal status of any country, territory, city, or area, or of its authorities, or concerning the delimitation of its frontiers or boundaries.
*

In West Africa MC is common and the prevalence of HIV is low ( Figure 1 and Figure 2A and 2B), while in southern Africa the reverse is true. Using these values in our static model ( Equation 1), circumcising all uncircumcised men in Africa would reduce the incidence of HIV as shown in Figures 2C and 2D. When incidence is measured as an absolute reduction in the number of incident infections expressed as proportion of the adult population, the potential reduction in HIV transmission is greatest in southern Africa ( Figure 2); when measured as a reduction in the total number of incident cases, the region of greatest impact extends to parts of East and Central Africa, as well as Ethiopia and Nigeria, which have large populations ( Figure 2). In South Africa alone, increasing MC coverage has the potential to avert up to 174,000 new infections each year ( Table 1).

While useful and immediate, the results in Figure 2 do not provide estimates of the differential impact of MC on the risk of infection in circumcised and uncircumcised men and in women, and do not capture the temporal dynamics of the impact on incidence, prevalence, and deaths. Using a two-sex model ( Equation 7), we first determine the ratio of the prevalence of HIV in women to men at the steady state. In South Africa, if no men were circumcised, we predict that 52% ± 4% of HIV-positive adults would be women, whereas if all men were circumcised this proportion would increase to 58% ± 4%. Using a three-group model ( Protocol S1), the expected prevalence of HIV in circumcised men is close to 80% of that in uncircumcised men regardless of the overall proportion of men who are circumcised (see Figure S3). We note that these results assume random mixing and use a simplified description of heterogeneity in risk behaviour; where this assumption is not true the relative prevalence in the two groups of men may be different. Whereas MC provides greater long-term benefits to circumcised than to uncircumcised men, and to men than to women, the differences in the steady-state prevalence among the three groups are not large and suggest that the use of a simplified one-group model will be acceptable.

In developing a one-group model to fit country-level data and to project changes in HIV incidence, prevalence, and deaths, we note ( Equation 6) that reducing female-to-male transmission by a proportion *π,* without changing male-to-female transmission, is equivalent to reducing transmission in the one-group model by factor of 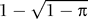. In particular, if MC reduces female-to-male transmission by 60%, then this is equivalent to reducing transmission in the one-group model by 37%, so that MC would have a population-level impact equivalent to an intervention that reduces transmission in both directions by 37%.

**Figure 3:**
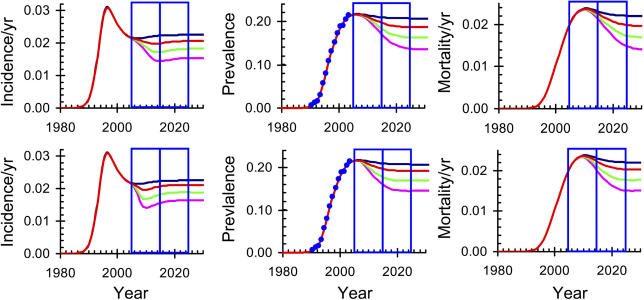
Time Trends in the HIV Incidence, Prevalence, and Related Mortality in South Africa if the Proportion of Circumcised Men Remains Constant or Is Increased to Full Coverage (over Five or Ten Years) (A) HIV incidence (B) HIV prevalence (C) HIV-related mortality in South African adults assuming that full coverage of MC is reached in 2015. The model is fitted to the blue data points in (B). (D–F) Repeat (A–C) but assuming that full coverage is reached in 2010 (see Protocol S1). The blue lines give the projected values excluding the impact of MC or other additional interventions. The red, green, and pink lines give the projected values assuming that MC reduces female-to-male transmission by 32%, 60%, and 76%, respectively, corresponding to the estimated reduction and 95% confidence intervals observed in the RCT of MC [ 9]. On all graphs, the blue boxes on the left mark the period 2005 to 2015, the blue boxes on the right mark the period 2015 to 2025.

We fitted this model to UNAIDS estimates of HIV prevalence over time for each country in sub-Saharan Africa to determine the model parameters, as illustrated for South Africa in Figure 3. The centre panel ( Figure 3B) shows the prevalence of HIV in South Africa obtained from national antenatal clinic data [ 38], adjusted to match UNAIDS estimates for the adult population [ 12]. We fit the model to the prevalence data, from which incidence ( Figure 3A) and deaths ( Figure 3C) follow directly. We then allow MC coverage to increase from 35% in 2005 to full coverage in 2015 and in 2010 (see Protocol S1) as illustrated in the top three panels and bottom three panels of Figure 3, respectively.

We first examine the impact on HIV if full coverage of MC is reached in 2015. The incidence of HIV infection responds immediately to the intervention ( Figure 3A) and by 2015 is close to its new asymptotic value. Prevalence responds more slowly and only approaches its new asymptotic value in 2025, while the reduction in mortality is slower still. In South Africa ( Figure 3 and Table 2), MC could avert 0.5 (0.3−1.0) million infections but only 0.1 (0.0−0.1) million deaths in the first decade of the program, 0.9 (0.5−1.8) million new infections and 0.7 (0.4−1.3) million deaths in the following decade, and 1.0 (0.5−2.0) million new infections and 1.2 (0.6−2.3) million deaths in the decade after that. The percentage reductions from the baseline scenario (with no increase in MC) as shown in Table 2 are much less in West Africa than in the other regions, but, because of the large population, the number of cases or deaths averted in West Africa is almost as great as in Central Africa, although still much less than in East and southern Africa. Over the 20 years from 2005 to 2025, in the whole of sub-Saharan Africa, MC could avert 5.7 million new cases and 3.0 million deaths, while reducing the number of people infected with HIV in 2025 by 4.1 million ( Table 2). We note that in South Africa the prevalence of HIV is high and the prevalence of MC is low, but the population is large, and approximately one-quarter of all infections prevented and deaths averted could be in South Africa.

**Table 2:**
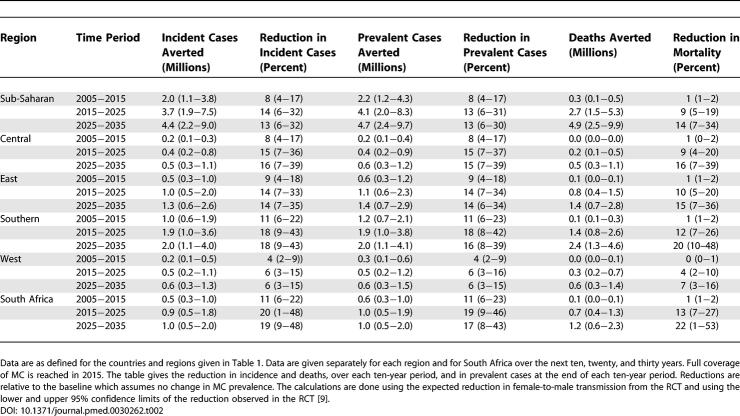
Impact of MC on HIV Incidence, Prevalence, and Related Deaths in Africa

Figure 3D– 3F repeats Figure 3A– 3C, but with the intervention being introduced twice as quickly and reaching full coverage in 2010. While the incidence declines much more rapidly ( Figure 3D and 3A, respectively), there is little change in the rate at which prevalence and deaths respond ( Figure 3E and 3B; Figure 3F and 3C; respectively), reflecting the low incidence and slow progression to death for people infected with HIV.

## Discussion

Our analysis uses a simple, parsimonious model to evaluate the potential impact of MC, and further empirical research is needed to support more detailed models. However, this analysis shows that MC could avert nearly six million new infections and save three million lives in sub-Saharan Africa over the next twenty years. Especially in southern Africa this could go some way to meeting the 2001 United Nations General Assembly Special Session on AIDS targets, the Millennium Development Goals, and the objectives set by bilateral donors, such as the US President's Emergency Plan for AIDS Relief.

Many questions remain to be answered. Better data are needed on the national prevalence of HIV in Africa, as well as on the associated uncertainties. UNAIDS gives plausibility bounds for estimates of national HIV prevalence that are typically about ±30% [ 12], so the absolute values of the estimates presented here are also uncertain to this extent, although the trends should be more reliable. Better data are also needed on the prevalence of MC in Africa and on the age at circumcision, preferably at the subnational level. Most of the currently available data on the prevalence of MC are several decades old, while several of the recent studies were carried out as adjuncts to demographic and health surveys and were not designed to determine the prevalence of MC [ 33]. Without such data it is difficult to make reliable estimates of the overall uncertainty in the potential impact of MC on HIV in Africa. Data are also needed on current circumcision practices, especially with regard to safety, on the acceptability of MC, on the cost of MC, and on the feasibility of making it available in places where it is not routinely done. A detailed study is needed of the cost effectiveness of MC as a way of managing the HIV epidemic in Africa using a dynamic model of transmission, accounting for the cost of MC and allowing for the savings that follow reductions in AIDS-related morbidity and the need for ART. In addition, this analysis is based on the result of just one RCT; it will be necessary for the results of that trial to be confirmed before it is clear how accurate these estimates of future infections are.

While the models presented here are a first step towards predicting the impact of MC on HIV in Africa, more detailed models are needed to explore the effect of MC on the age-specific incidence and prevalence of HIV among men and women and on the relative benefits of initially targeting men in certain age groups or in high risk occupations, such as truck drivers or mine workers.

Synergies with other potential interventions, including HIV vaccines, should also be explored, as well as possible synergies acting through the impact of MC on other sexually transmitted infections. Since *R*_0_ for HIV is on the order of 5–10, the 37% reduction in overall transmission associated with MC could make a significant contribution towards reaching the target of reducing *R*_0_ to 1 in the areas where few men are currently circumcised. Combined with other interventions to reduce transmission, this raises the possibility of reducing the prevalence of HIV to such low levels that it is no longer a major public health problem. The impact of MC in South Africa may also be mediated by its impact on other sexually transmitted infections; the results of the other two RCTs of MC, currently being conducted in Kenya [ 15] and Uganda [ 16] where the prevalence of other sexually transmitted infections may be different, should throw light on this.

The impact of MC on HIV should also be considered in the context of the increasing availability of ART. To the extent that ART reduces transmission, it will also reduce *R*_0_ and act synergistically with MC. Many studies have shown that ART leads to substantial declines in plasma viral load [ 39] and may reduce the risk of transmission for those on ART [ 40]. However, if people in Africa start ART late in the course of their HIV infection, the provision of ART is unlikely to reduce overall transmission significantly [ 41, 42].

As a cautionary note, increases in risk-taking behaviour among circumcised men could reduce the benefit of MC. The RCT [ 9] on which these models are based followed men for an average of 18 months, so that the effects of short-term behaviour changes have been accounted for. Community or population level studies of MC are now needed to determine the likelihood of behavioural disinhibition and to assess its impact on transmission in the long term.

While MC confers greater direct benefits on men than on women, women benefit indirectly through the reduction in the prevalence of HIV among their male sexual partners. Nevertheless, it is already the case that in Africa more women than men are infected with HIV [ 12], and additional methods that help to protect women, such as the development of effective vaginal microbicides, are still needed. A trial under way in Uganda [ 16] has been designed to measure the impact of MC on male-to-female transmission of HIV.

The earlier observational studies and the recent RCT all suggest that MC will have a long-term, population-level impact on HIV transmission. However, this assumption needs to be tested at the population level, and a large-scale, community-based programme to implement MC as widely as possible should be implemented and carefully monitored to determine the population level impact of MC directly.

This analysis makes it clear that MC could have an immediate impact on HIV transmission, but the full impact on prevalence and deaths will only be apparent about ten to fifteen years later. The reason is that circumcision averts infections some years into the future among people who would have died ten years later, on average. The same argument applies, of course, to other prevention methods because reductions in illness and death will only be manifested a decade or more after their introduction. The need to keep HIV-positive people alive through the provision of ART remains the most immediate priority while ways are found to reduce transmission using MC and other interventions.

## Supporting Information

Figure S1 The Increase of MC in South Africa Starting from a Coverage of 35% and Reaching Full Coverage in 2015 (Red Line) and 2010 (Blue Line) (365 KB JPG)

Figure S2 The Percentage of All HIV Cases That Occur in Women, as a Function of Circumcision Parameters (A) Circumcision is assumed to have no protective benefit for women ( *π _f_*= 0).
(B) Circumcision coverage is 100% ( *χ* = 1). Other parameters were chosen with reference to the South African HIV epidemic as discussed in the main text: *δ* = 0.102 yr ^−1^, *cφ _m_* = 0.52 y ^−1^, *cφ _f_* = 1.04 y ^−1^.
(2.7 MB JPG)

Figure S3 Ratio of the Prevalence of HIV in Circumcised to Uncircumcised Men at the Steady State Assuming an Average Infectious Period of 9.8 Years The relationship is almost completely independent of the circumcision coverage, *χ,* and the possible protective effect for women, *π _f_*. Other parameters: *χ* = 1, *π _f_* = 0, *cφ _m_* = 0.52/y, and *cφ _f_* = 1.04/y.
(490 KB JPG)

Figure S4 Comparison of Three-Group (Blue Line) and the Equivalent One-Group (Red Line) Models Parameter values: *χ* = 0.35, *π _m_*= 0.60, *π _f_* = 0, *cφ _m_* = 0.52/y, and *cφ _f_* = 1.04/y, *δ* = 0.102/y, and *p* = 0.29.
(7.8 MB TIF)

Figure S5 Absolute Difference between the Percent Prevalence as Predicted by the One-Group (Collapsed) Model and the Three-Group Model for Different Levels of MC Coverage The lines give different levels of protective efficacy *π _m_*: green, 0.76; red, 0.60; black, 0.32. Other parameters are the same as in Figure S4.
(431 KB JPG)

Figure S6 Force of Infection, Scaled to One at Zero Prevalence, as a Function of Prevalence The red line is estimated from survey data for Carletonville, South Africa. The green line assumes that the risk declines exponentially with prevalence, the blue line that the risk has the same value for all those who are at risk as assumed in the EPP model. The curves are scaled to pass through the point where the curve based on the Carletonville data passes through the prevalence among men in the survey (21%).
(587 KB JPG)

Protocol S1 MC and HIV in Africa (5.1 MB DOC)

## Abbreviations

ART: anti-retroviral therapy
MC: male circumcision
RCT: randomized control trial
