# Supplementary material for: The Potential Impact of Male Circumcision on HIV in Sub-Saharan Africa
Source: PLoS Med. 2006 Jul 11;3(7):e262. doi: 10.1371/journal.pmed.0030262 (PMC1489185; doi:10.1371/journal.pmed.0030262)
Supplement: Supplementary file 1 [file pmed.0030262.sd001.doc]

# Male circumcision and HIV in Africa

# Mathematical note

The formal definition of a convolution (main text; Equation 14) is

$$A\left(t\right)\otimes B\left(t\right)\equiv\int_{-\infty}^{+\infty} A\left(\tilde{t}\right)B\left(t-\tilde{t}\right)d\tilde{t}$$

# Rate of expansion of male circumcision

We assume that the coverage of MC increases logistically with time. The analytical expression for coverage is then

$$\chi=\chi_{0}+\left(1-\chi_{0}\right)\frac{\text{e}^{\alpha\left(t-t_{0}\right)}}{\text{1+e}^{\alpha\left(t-t_{0}\right)}}$$

where *χ*_0_ is the initial prevalence of MC, *t*_0_ is the time at which the logistic term reaches 0.5, and *α* determines the rate at which the coverage of MC increases. For the ten year expansion we set *t*_0_ = 2010 and *α* = 0.6/year while for the five year expansion we set *t*_0_ = 2007.5 and *α* = 1.2/year giving the coverage as a function of time, illustrated for South Africa in Figure S1.

Figure S1. The increase of MC in South Africa starting from a coverage of 35% and reaching full coverage in 2015 (red line) and 2010 (blue line).

# Separating contact and transmission parameters

We focus on interventions that reduce the probability of transmission without changing sexual behaviour. The key result (Equation 6 in the main text) is that *R*_0_ can be expressed as

$$R_{0}=f\left(\varphi\right)g\left(c\right)$$

where *φ* depends on those parameters that determine the risk of infection given contact with an infected person and *c* depends on those parameters that determine the number of sexual contacts per unit time. For interventions that reduce transmission probabilities while not affecting sexual behaviour, changes in the epidemic can be estimated from changes in the transmission probabilities without reference to the (unchanging) sexual behaviour. An absolute value of *R*_0_ during the intervention can be estimated by multiplying the relative change by an empirical estimate of the pre-intervention *R*_0_, which implicitly includes the complexities and heterogeneities of sexual behaviour.

## Random mixing

If the intervention leads to changes in sexual behaviour, at either the individual or population level, then further analysis is required. Such scenarios have been considered in detail in previous work [1-6]. If only a fraction of the population is covered by the intervention (such as circumcision or vaccination), then the decoupling argument assumes that individuals mix randomly with respect to intervention coverage status. If interventions are targeted at specific risk groups the contact rates and probability of transmission given contact are not independent and cannot be decoupled. If higher-risk groups are targeted successfully, then results from the decoupled analysis will place a conservative lower bound on the benefits of the intervention.

## Differential impact of male circumcision on different groups

Because of the asymmetry of HIV transmission between men and women, prevalence among women is generally higher than prevalence among men in heterosexual epidemics [7]. Higher levels of circumcision could increase this asymmetry in transmission, and increase the imbalance in prevalence between men and women. Circumcision will reduce the prevalence of infection among all men but will have a greater impact on those that are circumcised. In the main text we calculate the impact of MC averaged over women, circumcised men, and uncircumcised men, and we make separate estimates of the relative impact on men and women. Here we analyse a model that explicitly divides the population into women, circumcised men, and uncircumcised men in order to confirm the validity and test the accuracy of the results obtained in the main text. We employ a simple model formulation for analytic tractability bearing in mind that real-world complexities, including heterogeneity in risk and mixing patterns, could affect the results.

## The three-group model

The disease process is modelled using an SI (susceptible-infected) framework. Infected individuals die at per capita rate *δ*, but we assume constant population size for each group so that people who die are replaced immediately by susceptible people of the same type. Each individual is assumed to have heterosexual contacts at an effective contact rate *c*, and women divide their contacts among circumcised and uncircumcised men in proportion to their frequency in the population. We model transmission using frequency-dependent incidence, such that the incidence rate in each group is the product of the number of susceptible people in the group, their contact rate with members of other groups, the probability of infection given contact with members of other groups, and the prevalence of infection in the other groups. This model is a mechanistically accurate depiction of partnership-based HIV transmission, particularly for populations with frequent partner change [8].

Let *S_j_* and *I_j_* be the numbers of susceptible and infected individuals in each population group where *j = u, c* or *f* represent uncircumcised men, circumcised men, and women, respectively, and let *N_j_ =S_j_* + *I_j_* be the total number in each group. The three-group model can then be written:

$$\frac{dS_{u}}{dt}=\delta I_{u}-c \varphi_{m} S_{u}\frac{I_{f}}{N_{f}}$$

$$\frac{dI_{u}}{dt}=c \varphi_{m} S_{u}\frac{I_{f}}{N_{f}}-\delta I_{u}$$

$$\frac{dS_{c}}{dt}=\delta I_{c}-c \varphi_{m}(1-\pi_{m}) S_{c}\frac{I_{f}}{N_{f}}$$

$$\frac{dI_{c}}{dt}=c \varphi_{m}(1-\pi_{m}) S_{c}\frac{I_{f}}{N_{f}}-\delta I_{c}$$

$$\frac{dS_{f}}{dt}=\delta I_{f}-c \varphi_{f}Sf\left(\left(1-\chi\right)\frac{I_{u}}{N_{u}}+\chi\left(1-\pi_{f}\right)\frac{I_{c}}{N_{c}}\right)$$

$$\frac{dI_{f}}{dt}=c \varphi_{f}Sf\left(\left(1-\chi\right)\frac{I_{u}}{N_{u}}+\chi\left(1-\pi_{f}\right)\frac{I_{c}}{N_{c}}\right)-\delta I_{f}$$

Here *δ* is the disease-induced mortality, *c* is the effective contact rate, *χ* is the proportion of men that are circumcised, *φ_m_* is the probability per contact of female-to-male transmission, and *π_m_* is the proportional reduction in this probability if a man is circumcised; *φ_f_* and *π_f_* are the corresponding quantities for male-to-female transmission.

To simplify the model, we convert the equations to proportions, introducing new state variables $i_{u}=I_{u}/N_{u},$ $i_{c}=I_{c}/N_{c},$ and $i_{f}=I_{f}/N_{f}$. The three-group model is then represented by the following system of equations:

$$\frac{di_{u}}{dt}=c \varphi_{m} (1-i_{u}) i_{f}-\delta i_{u}$$

$$\frac{di_{c}}{dt}=c \varphi_{m} (1-i_{c})(1-\pi_{m}) i_{f}-\delta i_{c}$$

$$\frac{di_{f}}{dt}=c \varphi_{f} (1-i_{f})\left(\left(1-\chi\right) i_{u}+\chi\left(1-\pi_{f}\right) i_{c}\right)-\delta i_{f}$$

We found the endemic equilibrium of this system using *Mathematica* *5.0* (Wolfram Research, Champaign IL), and calculated relative measures of the long-term burden of HIV in different groups under different circumcision scenarios. The resulting expressions are too complex for direct interpretation, but are available from the authors upon request. The proportion of prevalent cases that are women agrees precisely with Equation 7 of the main text (derived from a simpler two-group model) when *χ* = 0 or 1, and differs by less than 1.3% at intermediate MC coverage.

Figure S2. The percentage of all HIV cases that occur in women, as a function of circumcision parameters. In (a) circumcision is assumed to have no protective benefit for women (*π_f_* = 0). In (b), circumcision coverage is 100% (*χ* = 1). Other parameters were chosen with reference to the South African HIV epidemic as discussed in the main text: *δ* = 0.102 yr^–1^, $c\varphi_{m}$= 0.52 yr^–1^, $c\varphi_{f}$ = 1.05 yr^–1^.

## The impact of MC on men and women

Figure S2 shows the proportion of all prevalent HIV cases that are women under two scenarios: assuming that MC has no effect on male-to-female transmission (Figure S2a), and assuming that circumcision coverage is complete but allowing for an effect on female-to-male and male-to-female transmission (Figure S2b). Figure S2a shows that the proportion of cases that are women could only increase to 70% if all men are circumcised and MC reduces female-to-male transmission by 88% or more (*π_m_* ≥ 0.88), or if at least 61% are circumcised and MC reduces female-to-male transmission by 99% (*π_m_* = 0.99). Figure S2b shows that, with 100% MC coverage, the proportion of prevalent HIV cases that are women could fall to 40% only if MC reduced male-to-female transmission by at least 88% (if female-to-male transmission is not reduced at all), or if MC reduced male-to-female transmission by 99% and female-to-male transmission by 78% or less.

If the per contact probability of HIV transmission from uncircumcised men to women is twice that from women to uncircumcised men (i.e. *φ_f_* /*φ_m_* = 2.0), then in a population with no male circumcision (*χ* = 0) we calculate that 52% of HIV cases will be women (Figure S2a). In a wholly circumcised population (*χ* = 1), if circumcision provides 60% protection to men (*π_m_* = 0.60) and no protection to women (*π_f_* = 0), then the proportion of HIV cases who are women will increase to 58%. In this scenario (*χ* = 1, *π_m_* = 0.60), even if circumcision reduces male-to-female transmission by 50% (*π_f_* = 0.5), women will still comprise 55% of HIV cases (Figure S2b).

## The impact of MC on circumcised versus uncircumcised males

Figure S3 shows the impact of MC on the ratio of the prevalence in circumcised to uncircumcised men as a function of the reduction in transmission. If circumcision reduces female-to-male transmission by 60%, HIV prevalence among circumcised men is still expected to be about 78% of that among uncircumcised men, and it is only when the reduction in transmission is greater than about 80% that the difference in the prevalence in the two groups is substantial. The chronic nature of the infection and the interconnectedness of the population both act to average out the risk in the long term. Note, however, that this prediction could change if circumcised and uncircumcised men were in different communities and sexual mixing among communities was limited.

Figure S3. Ratio of the prevalence of HIV in circumcised to uncircumcised men at the steady-state assuming an average infectious period of 9.8 years. The relationship is almost completely independent of the circumcision coverage, *χ*, and the possible protective effect for women, *π_f_*. Other parameters: *χ* = 1, *π_f_* = 0, *cφ_m_* = 0.52/yr and *cφ_f_* = 1.04/yr

## Collapsing the three-group model to a one-group model

In the main text, we fit trends and make projections based on country and regional HIV prevalence data. Because separate time series do not exist for females, uncircumcised males, and circumcised males, it is unnecessarily cumbersome to use the three-group model so we collapse the three-group model to a one-group SI model. Here we carry out simulations to determine the levels of error that may introduced by this procedure.

To represent the effects of circumcision in the collapsed model, we use the approximations discussed in the main text to replace Equations − by a single equation governing the proportion, *i*, of all individuals who are infected:

$$\frac{di}{dt}=c\sqrt{\varphi_{f}\varphi_{m}\left(1-\pi_{m}\chi\right)\left(1-\pi_{f}\chi\right)}\left(1-i\right)i-\delta i$$

To compare the two models quantitatively, we choose basic parameters to fit the data for the South African epidemic, as described in the Methods section of the main text. Fitting an exponential trend line to the South Africa prevalence data gives *r* = 0.55 ± 0.16/year (best estimate ± standard error), which corresponds to an intrinsic doubling time of *d* = 1.26 ± 0.37 years. The life expectancy of people infected with HIV and without access to ART, standardized to a mean age at infection of 27 years, is *τ* = 9.8 ± 0.5 years [9,10], so the average mortality rate is 0.102 ± 0.005/year. We then calculate *R*_0_ = *r*/*δ*+1 [11], yielding the value 6.4 ± 1.6 using Monte Carlo simulation to estimate the standard error in the result. Since an estimated *χ* = 0.35 ± 0.10 of South African men are circumcised (Table 1) and MC reduces female-to-male transmission by *π_m_*=0.60 (0.32−0.76), the value of *R*_0_ in the absence of circumcision would be $\left(r/\delta+1\right)/\sqrt{1-\chi\pi_{m}}$ or 7.2 ± 1.8. We relate this empirical estimate of *R*_0_ to the expression derived from the two-sex model, $R_{0}=c\sqrt{\varphi_{f}\varphi_{m}}/\delta$ (Equation 6 in the main text), to estimate male-to-female and female-to-male transmission rates. Taking *φf*/*φm* to be 2.0 ± 0.5, we find *cφm* = 0.52 ± 0.16/year and *cφf* = 1.05 ± 0.29/year.

Figure S4. Comparison of three-group (blue line) and the equivalent one-group (red line) models. Parameter values: *χ* = 0.35, *π_m_* = 0.60, *π_f_* = 0, *cφ_m_* = 0.52/yr and *cφ_f_* = 1.04/yr, *δ* = 0.102/year and *ρ* = 0.29.

Finally, for this model comparison we scale the endemic prevalence using the Epidemiological Projection Package (EPP) method [12] assuming that a fraction *γ* of the population is at risk for HIV infection, while the remaining 1 − *γ* are at zero risk. For *R*_0_ = 6.4 in the presence of 35% MC coverage, we choose *γ* = 0.29 to yield a steady state prevalence near 24.6%, the estimated steady state for South Africa (Table 1). We simulated the two models using Berkeley Madonna (Berkeley CA), and the resulting epidemic curves are shown in Figure S4.

The initial doubling times are 1.35 years for the one-group model, and 1.26 years for the three-group model, both close to the data-derived value of 1.26 years. The full three-group model predicts an endemic prevalence of 23.8% while the collapsed one-group model predicts an endemic prevalence of 24.5%. Thus the collapsed model estimate of endemic prevalence has an absolute error of less than 1% or a relative error of less than 3%.

For the South African epidemic, then, the one-group model reproduces the predictions of the three-group model well, though it slightly overestimates the steady-state prevalence. To explore the accuracy of the one-group model as an approximation to the three group model we varied the value of *π_m_*, the protective efficacy for female-to-male transmission due to MC (Figure S5). Introducing a protective effect for females (*π_f_* > 0) always decreases the difference between the models. For parameter ranges of interest to our investigation, output from the collapsed one-group model matches that of the full three-group model with reasonable accuracy (i.e. within a few percentage points). Because other uncertainties in the system far exceed this margin of error, we use the one-group model to fit the prevalence data and estimate the impact of MC.

Figure S5. Absolute difference between the percent prevalence as predicted by the one-group (collapsed) model and the three-group model for different levels of MC coverage. The lines give different levels of protective efficacy *π_m_*: green, 0.76; red, 0.60; black, 0.32. Other parameters are the same as in Figure S4.

# Decline of transmission with increasing prevalence

There is a distribution of sexual risk within all populations depending on gender, age and other social factors [13-16]. The EPP model [12] assumes that people are either at a fixed risk or zero risk and the model adjusts the size of the risk group to get the desired endemic prevalence. Here we make the more realistic assumption that risk varies continuously in the population. Since the functional form of the sexual risk distribution will, to some extent, determine the way in which incidence falls as prevalence rises, it will also determine the way in which the impact of MC varies with increasing efficacy. It must be the case that the average contact rate of uninfected people declines with prevalence. If this were not so then people at lower risk would have to be infected, on average, before those at higher risk.

Let us assume that there are some number of risk groups, each with characteristic contact rate *c_j_* (*j* = 1,…, *n*), and that whenever a person dies in a certain risk group a new person is recruited to the same risk group. This ensures that the proportion of people in each risk group remains constant over time whereas, in reality, the attrition of people in higher risk groups may reduce the overall risk of infection. This calculation will tend to provide a conservative estimate of the extent to which average risk among those who are still uninfected declines with prevalence. We denote the number of susceptible and infected individuals in group *j* as *S_j_* and *I_j_*, and the group size *N_j_* = *S_j_* + *I_j_*. If individuals choose partners at random (i.e. the fraction of contacts with individuals in group *k* is equal to the fraction of all contacts contributed by members of group *k*), then the dynamics of each risk group are [4,17]

$$\frac{dS_{i}}{dt}=\mu I_{i}-c_{i}\varphi S_{i}\sum_{j=1}^{n} c_{j}\frac{I_{j}}{N}$$

so that at the steady state

$$\frac{I_{i}}{I_{0}}=\frac{c_{i}\left(N_{i}-I_{i}\right)}{c_{0}\left(N_{0}-I_{0}\right)}$$

where we have chosen one of the groups, labelled 0, as the reference group. Let *i_j_* = *I_j_/N_j_* be the prevalence in group *j*, and *Ω_j_* be the odds for the prevalence in group *j*, then

$$\frac{\Omega_{i}}{\Omega_{0}}=\frac{c_{i}}{c_{0}}$$

Table S1. The number of sexual partners in the last month as reported by men in Carletonville, South Africa [18]. ‘Other’ refers to people who say that they have had no sexual partners in the previous twelve months.

Q401: How many times have you had sexual intercourse with anyone in the last 12 months (other than a regular partner)? This includes mistresses, girlfriends, casual partners, prostitutes, or somebody you met in a bar or at a special occasion. (If none, then skip Q402).

| No. partners | Frequency | No. partners | Frequency |
| --- | --- | --- | --- |
| 0 | 186 | 11 | 0 |
| 1 | 257 | 12 | 2 |
| 2 | 99 | 13 | 0 |
| 3 | 44 | 14 | 1 |
| 4 | 17 | 15 | 0 |
| 5 | 4 | 16 | 0 |
| 6 | 5 | 17 | 0 |
| 7 | 3 | 18 | 0 |
| 8 | 2 | 19 | 0 |
| 9 | 0 | 20 | 16 |
| 10 | 4 | Other | 1594 |

Q402: How many different people have you had sexual intercourse with in the last month (apart from your regular partner?)

To obtain an admittedly rough estimate of the relative risk of infection at different prevalences we use data from a survey in Carletonville, South Africa, of the number of sexual partners (excluding regular partners) reported by men in the previous month [18]. We make several simplifying assumptions. First, we assume that the number of partners reported in the survey is indicative of each individual’s habitual contact rate. Second, we assume that those who say that they have had a sexual partner in the last year but not in the last month are at the same risk as those who say that they have had one sexual partner in the last month (so that we combine the categories 0 and 1 in Table S1). Third we assume that those who say that they have not had a sexual partner in the last year are indeed at no risk of infection.

To determine the relationship between the prevalence and the average risk we first choose a value for the prevalence among those who have had one sexual partner in the last month, *i*_0_. We then calculate the prevalence in all other classes, *i_j_*, using Equation . Knowing the prevalence in each class we can calculate the overall prevalence

$$i=\frac{\sum_{j} i_{j}N_{j}}{\sum_{j} N_{j}}$$

We then calculate the transmission parameter for susceptible individuals in each group

$$\lambda_{j}=\varphi c_{j}\frac{\sum_{k} c_{k}i_{k}N_{k}}{\sum_{l} c_{l}N_{l}}$$

To obtain the average value of the transmission parameter for all those who are still susceptible we take a weighted average over the groups

$$\lambda=\frac{\sum_{j} \lambda_{j}\left(1-i_{j}\right)N_{j}}{\sum_{k} \left(1-i_{k}\right)N_{k}}$$

Because the overall prevalence, *i*, is uniquely determined by the group prevalences, *i_j_*, by Equations to , the average force of infection can be expressed as a function of the overall prevalence, *λ*(*i*). The normalized force of infection for a given prevalence is therefore *λ*(*i*)/*λ*(0). We repeat the above process for a range of values of *i*_0_ to generate a plot of this relationship (Figure S6).

Although the exponential curve is close to the curve based on the Carletonville data there are many assumptions and approximations involved in the estimation and the result should be treated as illustrative of the effect that variation in the risk of infection might have. However, the blue line must necessarily be conservative and in the absence of further data the exponential model seems reasonable.

Figure S6. Force of infection, scaled to one at zero prevalence, as a function of prevalence. The red line is estimated from survey data for Carletonville, South Africa. The green line assumes that the risk declines exponentially with prevalence, the blue line that the risk has the same value for all those who are at risk as assumed in the EPP model. The curves are scaled to pass through the point where the curve based on the Carletonville data passes through the prevalence among men in the survey (21%).

# References

1.	Anonymous (2004) Report on the Global AIDS Epidemic. Geneva: UNAIDS.

2.	McLean AR, Blower SM (1995) Modelling HIV vaccination. Trends in Microbiology 3: 458-462.

3.	Hyman JM, Li J (1997) Behavior Changes in SIS STD Models with Selective Mixing. SIAM Journal of Applied Mathematics 57: 1082-1094.

4.	Garnett G (1998) The influence of behavioural heterogeneity on the population level effect of potential prophylactic type 1 human immunodeficiency virus vaccines. Journal of the Royal Statistical Society Series A 161: 209-225.

5.	Blower S, Schwartz EJ, Mills J (2003) Forecasting the Future of HIV Epidemics: the Impact of Antiretroviral Therapies & Imperfect Vaccines. 5: 113-125.

6.	Smith RJ, Blower SM (2004) Could disease-modifying HIV vaccines cause population-level perversity? Lancet Infect Dis 4: 636-639.

7.	Williams BG, Gouws E, Colvin M, Sitas F, Ramjee G, et al. (2000) Patterns of infection: using age prevalence data to understand the epidemic of HIV in South Africa. South African Journal of Science 96: 305-312.

8.	Lloyd-Smith JO, Getz WM, Westerhoff HV (2004) Frequency-dependent incidence in models of sexually transmitted diseases: portrayal of pair-based transmission and effects of illness on contact behaviour. Proc Biol Sci 271: 625-634.

9.	CASCADE Collaboration (2000) Time from HIV-1 seroconversion to AIDS and death before widespread use of highly-active anti-retroviral therapy. A collaborative analysis. Lancet 355: 1131-1137.

10.	Morgan D, Mahe C, Mayanja B, Okongo JM, Lubega R, et al. (2002) HIV-1 infection in rural Africa: is there a difference in median time to AIDS and survival compared with that in industrialized countries? AIDS 16: 597-603.

11.	Anderson RM, May RM (1992) Infectious Diseases of Humans. Oxford.

12.	Ghys PD, Brown T, Grassly NC, Garnett G, Stanecki KA, et al. (2004) The UNAIDS Estimation and Projection Package: a software package to estimate and project national HIV epidemics. Sexually Transmitted Infections 80: 5-9.

13.	Carael M, Holmes K (2001) (editors) The multicentre study of factors determining the different prevalences of HIV in sub-Saharan Africa. AIDS 15: S1-S132.

14.	Garnett GP, Anderson RM (1996) Sexually transmitted diseases and sexual behavior: insights from mathematical models. J Infect Dis 174 Suppl 2: S150-161.

15.	Woolhouse ME, Dye C, Etard JF, Smith T, Charlwood JD, et al. (1997) Heterogeneities in the transmission of infectious agents: implications for the design of control programs. Proceedings of the National Academy of Sciences of the United States of America 94: 338-342.

16.	Schneeberger A, Mercer CH, Gregson SA, Ferguson NM, Nyamukapa CA, et al. (2004) Scale-free networks and sexually transmitted diseases: a description of observed patterns of sexual contacts in Britain and Zimbabwe. Sexually Transmitted Diseases 31: 380-387.

17.	Jacquez JA, Simon CP, Koopman J, Sattenspiel L, Perry T (1988) Modelling and Analysing HIV transmission: the effect of contact patterns. Mathematical Biosciences 92: 119-199.

18.	Williams BG, Gilgen D, Campbell CM, Taljaard D, MacPhail C (2000) The Natural History of HIV/AIDS in South Africa: a biomedical and social survey. Johannesburg, South Africa: CSIR.
